# Intracerebroventricular diphtheria toxin causes off-target toxicity in CD11b-DTR and wild-type mice, revealing limitations of DTR-based depletion studies

**DOI:** 10.3389/fnins.2026.1806305

**Published:** 2026-04-23

**Authors:** Jessica Irving, Peggy Rentsch, Sandy Stayte, Bryce Vissel

**Affiliations:** 1 Centre for Neuroscience and Regenerative Medicine, St. Vincent's Centre for Applied Medical Research, St Vincent's Hospital, Sydney, NSW, Australia; 2 Faculty of Medicine and Health, University of New South Wales, Kensington, NSW, Australia

**Keywords:** CD11b-DTR mice, diphtheria toxin receptor model, diphtheria toxin toxicity, microglia depletion, myeloid cell depletion

## Abstract

Diphtheria toxin receptor (DTR)–based depletion models are widely used to study microglial and macrophage function, yet the extent to which diphtheria toxin (DT) produces off-target effects remains incompletely defined. Here, we examined tolerability, behavioural outcomes, and cellular responses following intracerebroventricular (i.c.v.) DT administration in wild-type (WT) and CD11b-DTR mice. Mice received bilateral i.c.v. infusions of DT or vehicle over a 10-day period and were assessed for survival, motor and cognitive behaviour, myeloid cell changes, and neuropathology. Unexpectedly, DT induced dose-dependent mortality in WT mice, demonstrating that toxicity can occur independently of DTR expression. CD11b-DTR mice exhibited greater susceptibility, with reduced survival and the emergence of illness at lower DT doses. Behavioural testing revealed significant dose-dependent impairments in rotarod performance and Y-maze spontaneous alternation in both genotypes, while open-field mobility was largely preserved among animals. Region-specific analysis of myeloid cells in CD11b-DTR mice showed robust depletion in the midbrain at higher DT doses, whereas hippocampal cell numbers remained unchanged with marked morphological signs of activation. These findings indicate that DT-mediated myeloid cell responses vary across brain regions, potentially reflecting differential toxin exposure following ventricular delivery. Consistent with this, focal abnormalities in the brain—including ventriculitis, meningoencephalitis and spongiotic changes—were observed in a subset of clinically affected DT-treated animals, whereas peripheral organs were largely unremarkable and haematological changes were infrequent. Together, these data demonstrate that i.c.v. DT administration can induce mortality, behavioural dysfunction, and focal CNS pathology in both WT and CD11b-DTR mice, with transgene expression amplifying susceptibility. Our findings highlight the need for careful dose optimisation, appropriate DT-treated controls, and cautious interpretation of behavioural phenotypes when employing this model.

## Introduction

A widely used approach to study the role of microglia and macrophages is the use of the CD11b-diphtheria toxin receptor (DTR) transgenic mouse. In these mice the human DTR gene has been engineered under control of the endogenous Integrin Alpha M promoter (CD11b-DTR), rendering CD11b-expressing myeloid cells susceptible to diphtheria toxin (DT)-induced apoptosis. The system is predicated on the assumption that DT selectively targets DTR-expressing cells without off-target effects, as wild-type (WT) murine cells are considered highly resistant to DT due to differences in binding affinity of the mouse and human DTR (Mekada et al., 1982; Pappenheimer et al., 1982). Despite the widespread use of this model in the field, potential off-target effects of DT in both CD11b-DTR and WT mice have not been systematically examined which may affect how results obtained using these mice are interpreted.

To achieve depletion of myeloid cells in the central nervous system (CNS) in DTR transgenic mice, most studies have utilised a systemic, acute (1–3 days) course of DT via intraperitoneal (i.p.) injection (Frieler et al., 2015; Rubino et al., 2018; Peng et al., 2022; Bedolla et al., 2022). This approach induces rapid and profound depletion of microglia and infiltrating macrophages (~90%), but is also associated with several limitations, including systemic exposure to DT, potential activation of peripheral immune response, and variability in both the extent and duration of depletion reported across studies (Frieler et al., 2015; Rubino et al., 2018; Peng et al., 2022; Bedolla et al., 2022). Furthermore, repeated or high-dose administrations of DT have been reported to lead to the development of behavioural deficits in WT and DTR transgenic mice, raising questions surrounding the toxicity, specificity and reproducibility of DT in DTR-driven myeloid cell depletion models (Rubino et al., 2018; Peng et al., 2022).

In light of these concerns, this study employed a localised depletion strategy using intracerebroventricular (i.c.v.) administration of DT to reduce the potential for off-target effects associated with systemic exposure. Compared with i.p. administration, i.c.v. delivery permits lower doses to achieve CNS exposure while limiting peripheral distribution (Fahoum and Eyal, 2023). Accordingly, i.c.v. DT administration represents a rational approach to bias DT exposure toward the CNS while reducing, but not eliminating, potential for systemic off-target effects. Using this approach, we sought to define an i.c.v. DT dosing protocol that achieves central myeloid cell depletion while limiting off-target behavioural effects. However, we found that i.c.v. DT administration induced dose-dependent mortality and behavioural impairments in WT mice, and that this effect was exacerbated in CD11b-DTR mice. These findings indicate that DT toxicity may not be solely dependent on systemic exposure, underscoring the need for careful dose optimisation, appropriate controls, and cautious interpretation when utilising DTR-mediated depletion models.

## Methods

### Animals

The B6.FVB-Tg(ITGAM-DTR/EGFP)34Lan/J (CD11b-DTR/GFP) strain were originally obtained from the Jackson Laboratory (strain #006000). Only homozygous mice of both sexes were used in this study at 7–11 weeks old. For WT controls, C57BL/6JAusb mice were obtained from Australian BioResources. During experiments, mice were group housed (maximum 5 per cage) and maintained on a 12-h light/dark cycle with access to food and water *ad libitum*. Animal experiments were performed with approval of the Garvan Institute and St. Vincent’s Hospital Animal Experimentation Ethics Committee under approval numbers 20/10 and 23/13 in accordance with the Australian National Health and Medical Research Council animal experimentation guidelines and the local Code of Practice for the Care and Use of Animals for Scientific Purposes.

### Intracerebroventricular cannula implant

All surgeries were performed under ketamine (10 mg/mL; Mavlab) and xylazil (2 mg/mL; Troy Laboratories) anaesthesia (10 mL/kg). Mice were positioned in a stereotaxic frame (Kopf Instruments), and bilateral 0.08 mm burr holes were drilled at AP − 0.4, ML ± 1.0, relative to bregma and the dural surface. A bilateral ICV guide cannula (C235GS, 26G, Plastics1) was mounted in a stereotaxic holder and inserted to DV − 2.0. The cannula was secured with light-curing adhesive (iBond Universal, Kulzer; 20s cure) and dental cement (Tetric EvoFlow, Ivoclar; three layers, 60s each). A dust cap was attached, the incision sutured (Dynek), and mice placed in clean cages on heating pads. Postoperatively, mice were provided with sugared milk and 1 mL glucose/saline solution (6% w/v, s.c.) until original weight was recovered and cages were kept half on heating pads throughout the study.

### Diphtheria toxin administration

Lyophilised DT (from *Corynebacterium diphtheriae*, Sigma Aldrich) was reconstituted according to the manufacturer’s guidelines, aliquoted (2 μL), and stored at −40 °C. On the day of use, DT was diluted in sterile 1X PBS to 2–100 ng/μL. Following a 2-week surgical recovery period, bilateral i.c.v. infusions (0.125 μL/hemisphere; 0.25 μL total) of DT or vehicle control were delivered at 0.25 μL/min using a Hamilton syringe pump through the implanted guide cannula; CD11b-DTR mice were infused with PBS daily, 2 ng/μL daily, 10 ng/μL every second day (E2D) or 25 ng/μL E2D, and WT mice with PBS, 10 ng/μL or 100 ng/μL daily, over a 10-day administration period. To prevent reflux following the infusion, the cannulae was kept in place for 1 min post-infusion. On days where infusions and behavioural testing were both performed, infusions were conducted after behavioural testing to minimise potential stress-related effects on behaviour that could be induced by handling from the infusion process.

### Experimental design

To evaluate tolerability across dosage groups, animal conditions were monitored and recorded daily prior to DT administration. Mice that exhibited signs of illness, including severe weight loss (>20% pre-surgical body weight), ataxia, kyphosis or seizure were euthanised and recorded as mortalities once end-points were met for survival analysis. For behavioural assessments, animals were randomly assigned to testing on days 2, 4, 6 or 8 of DT treatment, with each mouse completing the open field, Y-maze and rotarod tasks on the same testing day. Data from all-time points were pooled within dosage groups to capture overall behavioural performance across the treatment course. All testing was performed by experimenters blinded to treatment in a quiet environment, with apparatus cleaned with 80% ethanol between subjects.

### Open field

Locomotor activity was assessed in an open field arena (273 × 273 × 203 mm; MedAssociates Inc) with clear plexiglass walls and white flooring, housed in a sound-attenuated chamber with uniform illumination. Mice were not habituated to the open field prior to testing. Mice were placed in the centre of the arena and allowed to openly explore the test box for 10 min, with total distance travelled assessed with Activity Monitor 7 (MedAssociates Inc) via infrared photobeam arrays.

### Y-maze

Spatial memory was assessed in a plexiglass Y-maze apparatus which consisted of three identical arms measuring 205 mm (L) × 50 mm (W) × 135 mm (H) positioned at 120° angles from each other. Mice were placed in the centre of the maze and were allowed to openly explore all arms for 5 min. Arm entries and spontaneous alternations were automatically recorded with ANYmaze Video Tracking System 6.33 (Stoelting Co.) Percentage of alternations were calculated as the number of alternations divided by the total number of arm entries (minus two to account for the first two arm entries) multiplied by 100.

### Rotarod

Motor coordination and balance were assessed using an accelerating rotarod (Ugo Basile; 5–40 rpm over 5 min). Latency to fall was measured across three trials per animal and results were averaged. All trials took place on the same day, and animals were given a 30-min rest in between test runs.

### Fluorescent immunohistochemistry

Anaesthetised mice were transcardially perfused with ice-cold saline followed by 4% paraformaldehyde. Whole brains were removed and post-fixed (24 h, 4 °C), cryoprotected in 30% sucrose (≥72 h), and coronally sectioned at 40 μm using a cryostat (Leica). Free-floating sections were blocked in 5% bovine serum albumin and 0.5% Triton X-100 in 1X PBS (1 h, room temp), incubated with rabbit anti-IBA1 (1:500, FUJIFILM Wako, 019–19,741, RRID: AB_839504; 72 h, 4 °C), washed, then incubated with Alexa Fluor 488 donkey anti-rabbit (Invitrogen, A32790, RRID:2762833; 24 h, 4 °C). Sections were counterstained with DAPI (1:1000, Invitrogen, D1306, 10 min, room temp), mounted on SuperFrost Plus slides (ThermoFisher Scientific), and cover slipped (Menzel-Glasser) with 50% glycerol mounting medium (Sigma Aldrich).

### Blood collection and histopathology

For blood collection, a micro capillary pipet (DWK Life Sciences) was inserted into the lateral canthus and blood collected from the retro orbital sinus. A minimum of 20 μL was collected in an EDTA coated tube (Microvette APT K2E, Sarstedt). Blood was then analysed with a VETSCAN HM5 hematology analyser (Zoetis) for complete blood cell counts. For histology, brain, spleen, liver, kidney, lung, heart and thymus tissue were harvested and preserved in 10% buffered formalin (Australian Biostain). Whole organ samples were sent to Cerberus Sciences (Melbourne, Australia) for tissue preparation and health analysis screening by a specialist veterinary pathologist.

### Cell quantification

Images were acquired using a Leica Thunder widefield microscope and computationally cleared with the Leica Application Suite X THUNDER imaging system (Leica) with a HC PL FLUOTAR L 20x/0.40 DRY objective. Double positive IBA1/DAPI immunostaining in the hippocampus (ML -1.4, AP -1.9, DV 1.75) and midbrain (ML 1.4, AP 3.7, DV -3.9) were manually quantified in single plane images with the ImageJ Cell Counter plug in (ImageJ, National Institutes of Health) with a 398x398μm field of view (FOV) in two representative coronal sections of both regions of interest. Counts from the two sections were averaged to yield a mean value per FOV for each region. Cell counts were performed by an investigator blinded to treatment.

### Statistical analysis

All statistical analyses were performed using Prism 10 (GraphPad Software). Survival and event-free probability analyses were conducted using Kaplan–Meier survival curves. Survival and event-free proportions were compared between experimental groups using the log-rank (Mantel–Cox) test and data were plotted as percent survival or event-free probability over time. For behaviour and cell quantification, Shapiro–Wilk tests were performed on all data sets to assess normality, before analysis with parametric tests. For normally distributed data, differences between means were assessed by one-way ANOVA with or without repeated measures, followed by Bonferroni *post-hoc* analysis. All quantification data are presented as mean ± standard error of the mean (SEM). For all statistical tests, *p* < 0.05 was assumed to be significant.

## Results

### Diphtheria toxin produces dose-dependent mortality in both WT and CD11b-DTR mice

**Figure 1:**
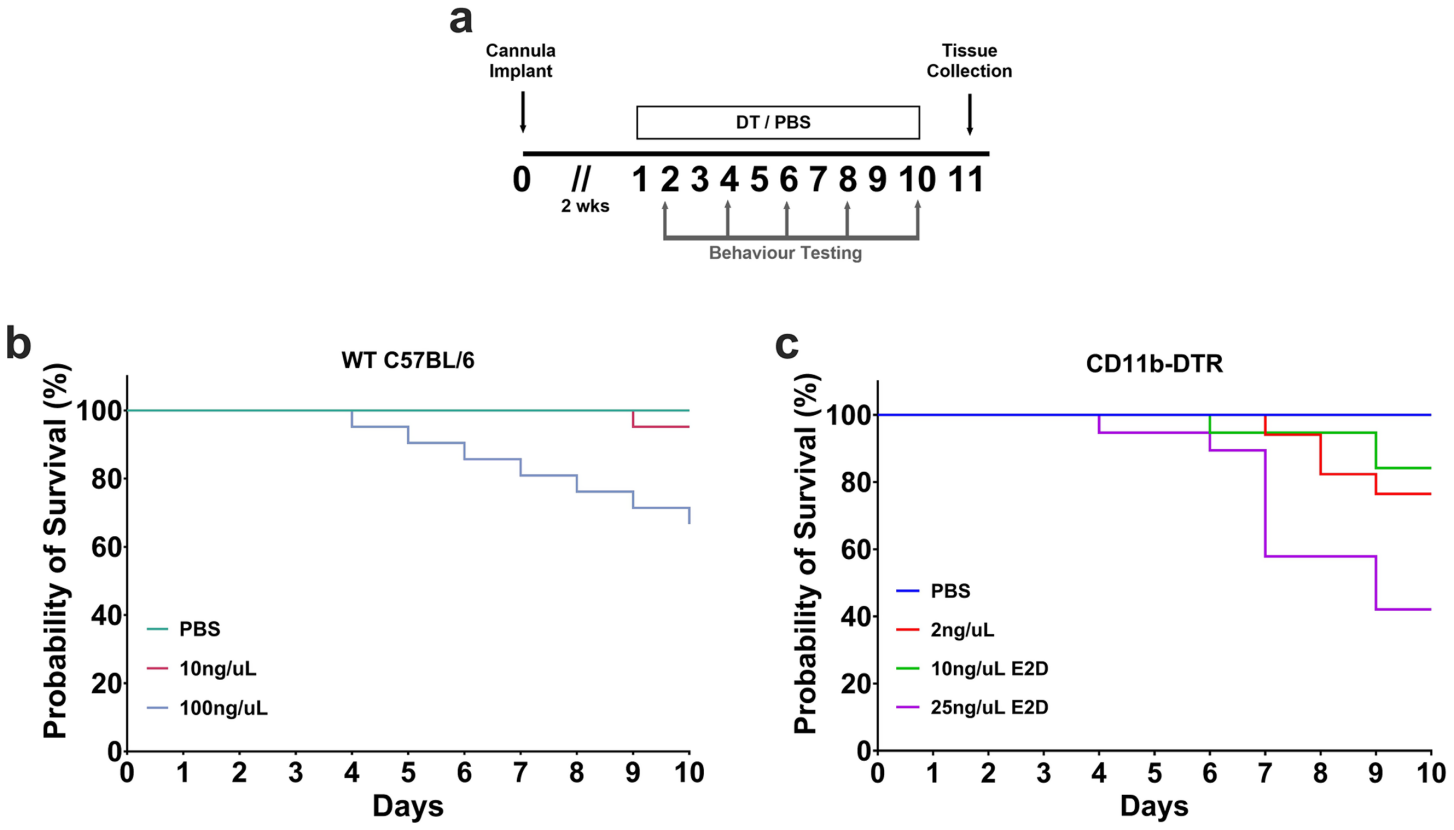
Kaplan–Meier survival analysis of DT-treated mice. **(a)** Experimental timeline. Mice were implanted with an i.c.v. cannula followed by a 2-week surgical recovery period. DT or PBS was administered beginning day 1, with behavioral testing conducted on days 2, 4, 6, and 8, and tissue collection on day 11. **(b)** Survival outcomes of WT C57BL/6 mice receiving daily PBS (*n* = 18) or DT (10 ng/μL (*n* = 21), 100 ng/μL (*n* = 21)), showing a dose-dependent decline in survival with high dose DT. **(c)** Survival outcomes of CD11b-DTR mice receiving PBS daily (*n* = 13) or DT (2 ng/μL daily (*n* = 17), 10 ng/μL E2D (*n* = 19), 25 ng/μL E2D (*n* = 19)). Higher DT doses were associated with reduced survival.

Given the initial purpose of this study was to optimise a dosage regimen of DT in CD11b-DTR mice, we evaluated multiple doses to assess tolerability and survival and employed WT C57BL/6 mice as controls. Therefore, in this study, mice were administered daily or E2D infusions of PBS or DT (2-100 ng/μL, 0.25 μL, i.c.v.) across a 10-day period, and survival was monitored (Figure 1a). Animals exhibiting signs of illness, including severe weight loss, ataxia, kyphosis or seizure (Incidence recorded in Supplementary material 1) were euthanised and recorded as mortalities once end-points were met. To first confirm there was no effect on survival related to the process of cannula implant and infusions, survival was monitored in PBS treated mice. Kaplan–Meier survival analysis revealed median survival was not reached in either WT or CD11b-DTR mice, with 100% of mice surviving to the study endpoint, confirming the administration procedure did not affect survival (Figures 1b, c). Unexpectedly, Kaplan–Meier survival analysis revealed a significant effect of DT dose on the survival distribution of WT mice (log-rank Mantel-Cox test [χ^2^ = 12.02, df = 2, *p* < 0.01], *n* = 18–21 per group, Figure 1b). Across dose groups, a 95 and 66% survival portion was recorded for 10 ng/μL and 100 ng/μL DT-treated WT mice, respectively, indicating a dose-dependent effect on mortality and that DT can affect WT animals independent of the lack of transgene expression.

Given that higher DT doses induced mortality in WT mice and CD11b-DTR mice are expected to be more sensitive to DT, CD11b-DTR animals were subsequently tested using lower doses of DT, as well as administration E2D. For CD11b-DTR mice, Kaplan–Meier survival analysis revealed a significant effect of dose on survival distribution (log-rank Mantel-Cox test, [χ^2^ = 17.24, df = 3, *p* < 0.001], *n* = 13–17 per group, Figure 1a). Mice treated with 25 ng/μL E2D had a median survival of 9 days (95%, Cl: 7) with a 42% survival portion, while the 2 ng/μL and 10 ng/μL E2D group had 76% and 84% survival portions, respectively. Together, these data demonstrate that CD11b-DTR mice exhibit greater susceptibility to DT-induced mortality than WT mice, as expected. This finding also highlights that survival itself may confound interpretation of all behavioural outcomes tested in this study and puts forward the notion that carefully defining dosing thresholds, in respect of WT tolerability, is necessary when utilising this model.

### WT and CD11b-DTR mice show no reduction in locomotor activity in the open field test

**Figure 2:**
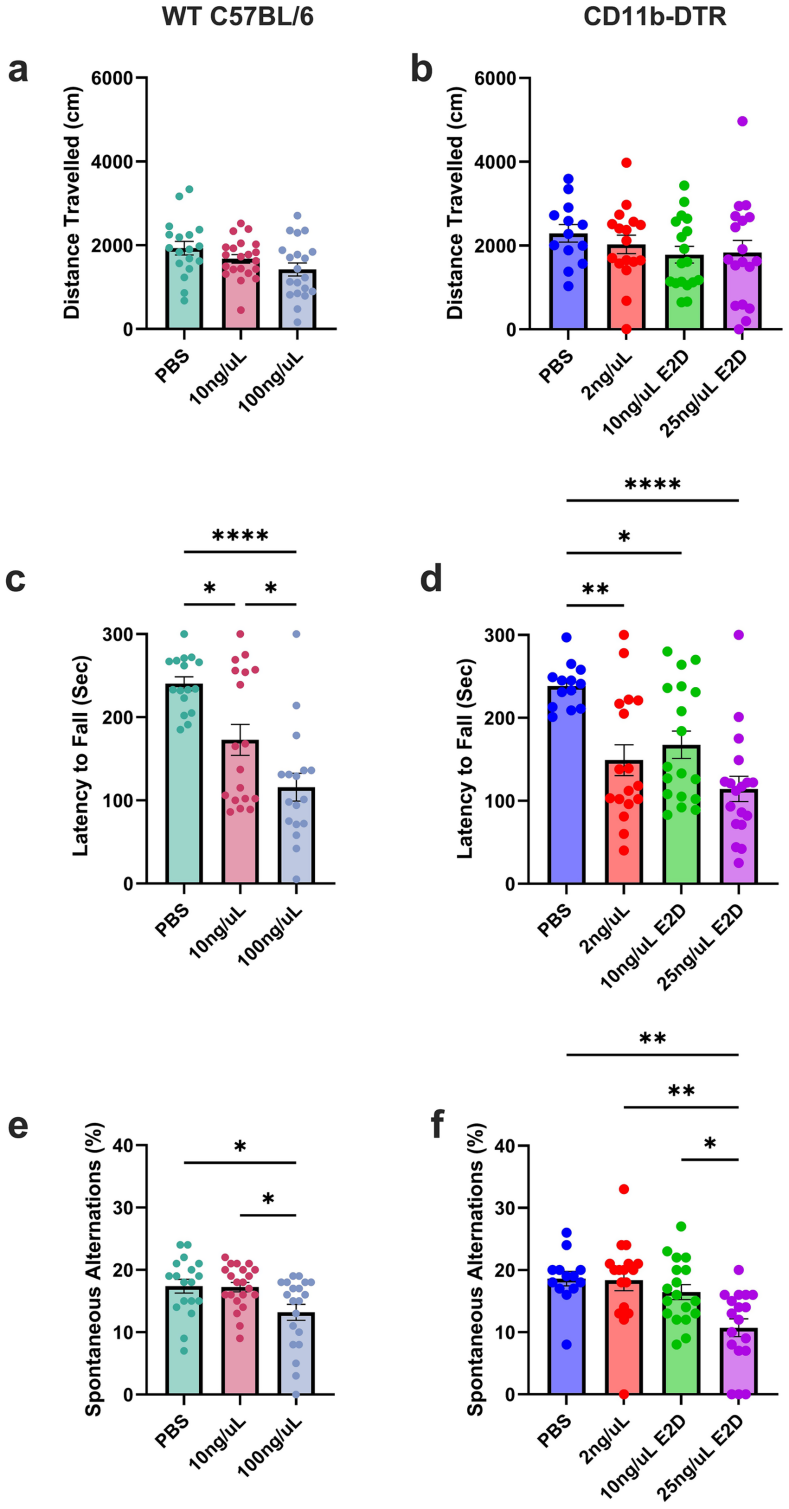
DT treatment induces behavioral abnormalities in CD11b-DTR and WT C57BL/6 mice. **(a)** WT and **(b)** CD11b-DTR mice treated with DT showed no changes in total distance traveled in the OFT. **(c)** WT mice displayed a dose-dependent fall latency on the rotarod relative to PBS controls and **(d)** CD11b-DTR mice treated with DT displayed a reduction in latency to fall, indicating motor impairment. **(e)** WT mice exhibited reduced working memory in the Y-maze at the highest dose of DT, while **(f)** CD11b-DTR mice treated with high dose DT displayed a reduction in spontaneous alternations when compared to controls. Data are presented as mean ± SEM. Each data point represents an individual animal. Statistical significance determined by one-way ANOVA with *post hoc* multiple comparisons; *p < 0.05; **p < 0.01; ****p < 0.0001.

As motor deficits have been previously associated with high dose DT treatment in both DTR and WT mice, we sought to assess the general motor behaviour of mice treated with varying doses of DT. Accordingly, WT and CD11b-DTR mice were assessed for locomotor activity in the Open Field Test (OFT). A one-way ANOVA revealed no significant effects in the total distance travelled among WT mice [*F*_(2,56)_ = 3.151, *p* = 0.0505, Figure 2a] and CD11b-DTR groups [*F*_(3,62)_ = 0.8425, *p* = 0.4758, Figure 2b]. While these results indicate that repeated DT exposure at the tested doses did not measurably reduce spontaneous locomotion in either genotype, the interpretation is constrained by attrition at higher doses: survival probability was reduced in both WT and CD11b-DTR groups (Figures 1b,c), and animals that met end-point criteria before behavioural testing could not be assessed. Thus, the OFT dataset is enriched for healthier survivors and may underestimate DT-related motor toxicity effects on locomotor behaviour. Consistent with this, the effect in WT mice approached significance (*p* = 0.0505), hinting at possible subtle alterations in activity at higher doses. Taken together, the data indicates general locomotor tolerance among survivors while reiterating dose-dependent mortality as a key determinant of overall tolerability.

### Motor coordination is impaired in a dose-dependent manner following DT treatment

To further evaluate motor coordination and balance beyond general locomotion, WT and CD11b-DTR mice treated with DT or vehicle were assessed on the accelerating rotarod. The rotarod task was utilised as another method of identifying motor deficits which may not have been detectable in the OFT. For WT animals, one-way ANOVA revealed a significant effect of treatment on latency to fall [*F*_(2,48)_ = 15.29, *p* < 0.0001; Figure 2c] with Bonferroni *post-hoc* analysis indicating that 10 ng/μL (*p* < 0.05) and 100 ng/μL (*p* < 0.0001) DT treated mice had lower latencies compared to vehicle controls. Furthermore, we observed a dose dependant effect of DT, with a significant decrease in time spent on the rotarod in 100 ng/μL DT treated animals compared to those receiving 10 ng/μL (*p* < 0.05). A one-way ANOVA revealed a significant change in the latency to fall among CD11b-DTR groups [*F*_(3,62)_ = 9.627, *p* < 0.0001; Figure 2d]. Bonferroni *post-hoc* analysis revealed that 2 ng/μL (*p* < 0.01), 10 ng/μL E2D (*p* < 0.05) and 25 ng/μL E2D (*p* < 0.0001) DT treated mice had significantly lower latency when compared to vehicle control. These results indicate that DT administration impairs motor coordination in both CD11b-DTR and WT mice. Importantly, deficits were evident at relatively low doses (2 ng/μL daily–10 ng/μL E2D) in CD11b-DTR mice, whereas WT animals exhibited impairments at higher doses (10–100 ng/μL daily), supporting the notion that, as expected, CD11b-DTR mice are overtly sensitive to developing motor deficits following DT, however our data also suggests DT itself can impair motor function independently of DTR expression.

### Higher doses of DT reduce spontaneous alternations in the Y-maze in WT and CD11b-DTR mice

To assess spatial working memory, we evaluated CD11b-DTR and WT mice in the Y-maze spontaneous alternation task. For WT animals, one-way ANOVA showed a significant treatment effect [*F*_(2,56)_ = 5.029, *p* < 0.05; Figure 2e] with Bonferroni *post-hoc* demonstrating reduced alternations in the high-dose group relative to vehicle controls (*p* < 0.05) and lower-dose animals (*p* < 0.05). Similarly, for CD11b-DTR mice one-way ANOVA revealed a significant effect of treatment on spontaneous alternation [*F*_(3,62)_ = 6.907, *p* < 0.05; Figure 2f]. Bonferroni *post-hoc* comparisons showed that the high-dose DT treatment significantly reduced alternation relative to controls (*p* < 0.01), as well as compared with the 2 ng/μL (*p* < 0.01) and 10 ng/μl E2D (*p* < 0.05) groups. Together, these findings demonstrate that both genotypes develop dose-dependent impairments in spatial working memory at higher doses of DT, in alignment with the survival and rotarod outcomes.

### Region-specific changes in IBA1 + cell number and morphology following DT treatment

**Figure 3:**
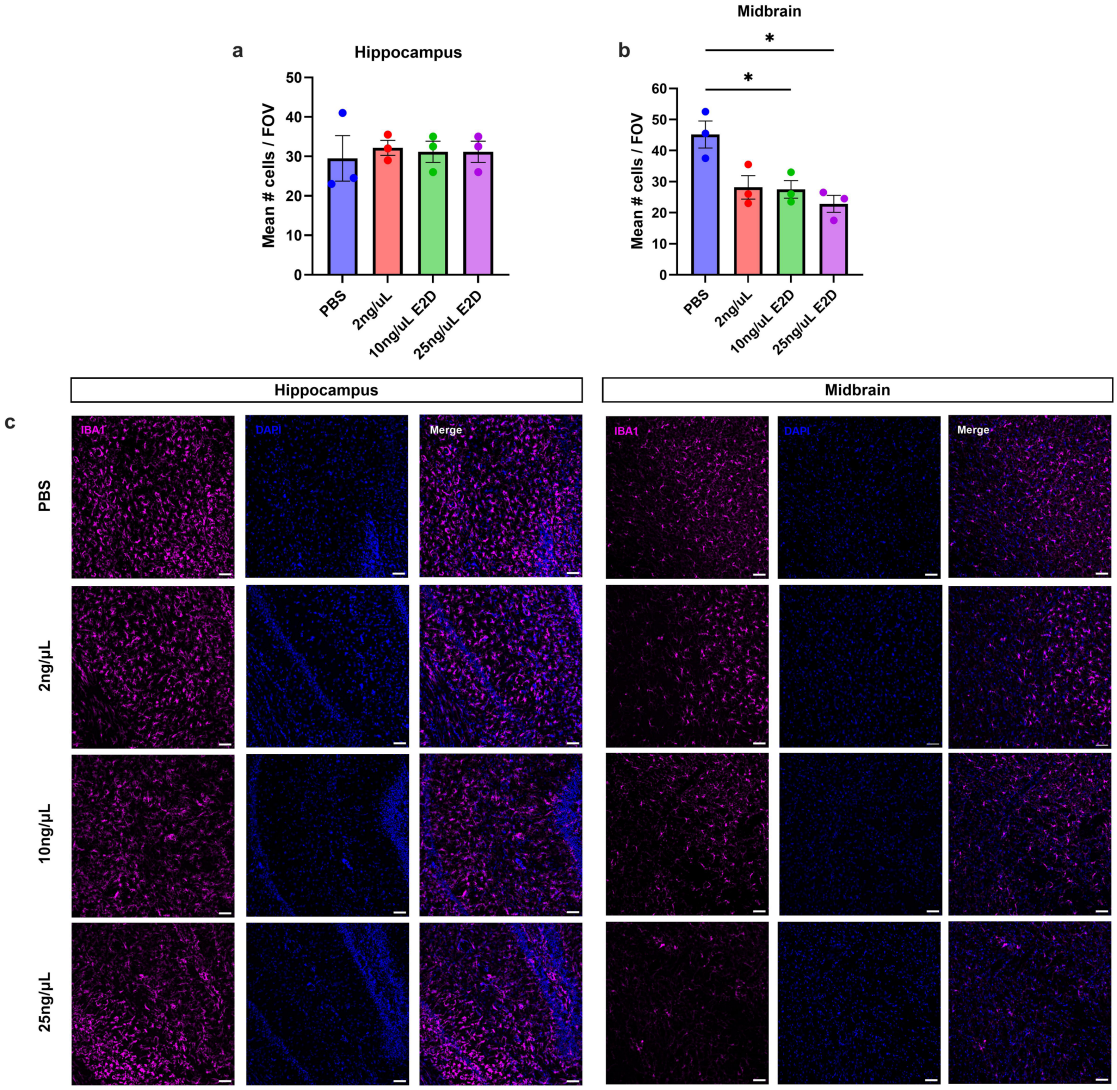
DT elimination efficiency in CD11b-DTR mice. **(a)** Quantification of IBA1^+^/DAPI^+^ cells revealed no significant change in the hippocampus across DT doses compared with PBS-treated controls in CD11b-DTR animals. **(b)** In the midbrain, a significant, dose-dependent reduction in IBA1^+^ cells was observed following 10 ng/μL E2D and 25 ng/μL E2D DT administration indicating robust depletion of myeloid populations in CD11b-DTR mice **(c)** Representative images showing IBA1 (magenta) and DAPI (blue) staining in the hippocampus and midbrain across treatment conditions. All values represent the mean ± SEM. *p < 0.05. Scale bar represents 50 μm.

To assess whether i.c.v. DT administration efficiently depleted myeloid cells under the DT dosing regiments tested, we quantified IBA1 + cells within the hippocampus (Figure 3a) and midbrain (Figure 3b) of CD11b-DTR mice. IBA1 was used as a pan-myeloid marker to quantify resident microglia and infiltrating macrophages. The hippocampus and midbrain were selected for analysis based on their relevance to spatial memory and motor function, respectively. One-way ANOVA revealed a significant effect of DT on IBA1 expression in the midbrain [*F*(3,8) = 7.908, *p* < 0.01], but no effect in the hippocampus [F(3,8) = 0.09555, *p* = 0.9603]. Bonferroni *post-hoc* analysis indicated that the 10 ng/μL E2D (*p* < 0.05) and 25 ng/μL E2D (*p* < 0.05) DT treatments produced a robust depletion of IBA1 + cells in the midbrain, while 2 ng/μL DT treatment had no significant effect (*p* = 0.052). Overall, these results indicate that DT administration effectively reduces IBA1 + cell populations at higher doses within the midbrain, but not the hippocampus. Observationally, IBA1 + cells possessed a more reactive morphology within the hippocampus (Figure 3c), with notable enlargement of cell bodies and retracted processes, suggesting the presence of inflammation which was not observed in the midbrain region. Interestingly, inspection of cell morphology in WT animals also indicated susceptibility to an inflammatory response in both 10 ng/μL an 100 ng/μL DT treated animals (Supplementary material 2), suggesting that DT exposure may provoke myeloid cell activation through DTR-independent mechanisms, which should be considered when interpreting DT-based depletion models.

### Higher doses of DT is associated with focal neuropathological and haematological abnormalities

For blood analysis and histology, animals which exhibited signs of illness, including severe weight loss, ataxia, kyphosis or seizure during the DT administration period were collected for targeted investigation of pathologies. Samples from both PBS-treated WT and CD11b-DTR animals showed non-significant diagnostic changes in both blood counts and histology analysis across all tested organs (Supplementary materials 3, 4). Contrastingly, high dose 100 ng/μL DT treated WT mice displayed focal pathological changes including occasional glial infiltrates within the ventricular lumen and mild acute ventriculitis and meningoencephalitis in the brain and mild lymphopenia in blood. Similarly, the 25 ng/μL E2D treated CD11b-DTR animal displayed mild acute meningoencephalitis with multifocal to diffuse spongiotic changes in the cerebral cortex and associate perivascular/neuropil oedema; several necrotic neurons were also present in this region. This animal also demonstrated more pronounced lymphopenia and mild neutropenia on hematology. Collectively, aside from these isolated observations, histology of all other organs was unremarkable.

## Discussion

Although DT-based depletion models have been widely used, the effects of DT exposure on baseline behaviour and cellular responses in both WT and CD11b-DTR mice have not been comprehensively characterised. Defining tolerance thresholds of DT in both CD11b-DTR and WT mice was therefore essential to assessing the reliability and interpretability of experiments that rely on DTR-mediated myeloid cell depletion. In the present study, we are the first to report on dose-dependent mortality and the development of motor and cognitive deficits in CD11b-DTR mice following i.c.v. DT administration and further identify that WT mice were also susceptible to mortality and behavioural abnormalities, suggesting off-target effects seen in the CD11b-DTR model may not be entirely dependent on transgene expression.

Survival outcomes were first examined in WT mice to establish baseline tolerance to DT exposure. We observed a clear dose-dependent reduction in survival, demonstrating that DT administration can exert toxicity independently of transgene expression. High-dose DT treatment was poorly tolerated, with significant mortality and the emergence of severe behavioural abnormalities, including ataxia-like movements, kyphosis, and seizures, which represented survival endpoints in this study. These findings align with prior reports by Peng et al. (2022), who observed poor health and subsequent death in WT mice treated with high dose DT (3× 1.5–2.0 μg i.p.) and the development of significant weight loss, ataxia-like behaviours and decline in latency during rotarod assessment, reinforcing that DT toxicity alone is sufficient to induce neurological dysfunction.

Given that survival outcomes revealed dose-dependent toxicity, we next examined behavioural performance in surviving WT animals to assess whether subtler functional impairments were evident. Using the OFT, rotarod, and Y-maze, we evaluated general locomotion, motor coordination, and spatial working memory, respectively, across DT-treated groups. In the OFT, no significant reduction in locomotor activity was detected, although a trend toward reduced movement was observed at higher DT doses, suggesting that gross locomotion may be relatively preserved among survivors. However, this interpretation may also be limited by mortality-driven attrition at higher doses. By contrast, the rotarod provided a more sensitive measure of motor function, revealing significant dose-dependent deficits in DT-treated WT mice, consistent with toxin-induced motor impairment. Likewise, the Y-maze revealed a dose-dependent decline in spontaneous alternation, with high-dose DT-treated WT groups showing reduced spatial working memory relative to controls. These findings confirm that DT exposure alone can produce motor and cognitive impairments independent of transgene expression.

In CD11b-DTR mice, DT administration produced similar but more pronounced effects. Survival analysis revealed a strong dose-dependent reduction in viability, with high-dose DT resulting in fewer than half of animals surviving to endpoint. Lower doses yielded intermediate survival rates, suggesting only a narrow margin between partial tolerability and lethality. Consistent with this increased vulnerability, CD11b-DTR mice displayed ataxia-like behaviours, kyphosis, and seizures, mirroring the phenotype observed in WT mice but emerging at lower doses. Behaviourally, DT-treated CD11b-DTR mice exhibited significant dose-dependent motor and cognitive impairments. On the rotarod, performance declined at lower DT doses compared to WT animals, confirming that transgene expression enhances susceptibility to DT-mediated toxicity. OFT analysis showed no significant reduction in locomotor activity across treatment groups, although subtle trends toward hypoactivity were observed at higher doses. In the Y-maze, DT-treated CD11b-DTR mice demonstrated dose-dependent impairments in spontaneous alternation, with high-dose groups exhibiting significantly reduced spatial working memory compared with controls. These findings indicate that DT exposure produces both motor and cognitive dysfunction in CD11b-DTR mice, with transgene expression amplifying vulnerability to DT-mediated neurotoxicity. Notably, Wang et al. (2020) reported effective microglial depletion using chronic i.c.v. DT administration (2 ng/μL daily for 35 days) in CD11b-DTR mice. However, as mortality or behavioural abnormalities were not assessed, direct comparison of tolerability is limited. In contrast, our findings demonstrate that measurable deficits in the rotarod and mortality are associated with an acute treatment course at this dose, suggesting that adverse effects may not be fully captured in paradigms where functional outcomes are not systematically evaluated, or may reflect discrepancies in monitoring or reporting.

The comparable behavioural abnormalities and mortality observed in both WT and CD11b-DTR mice indicate that DT toxicity is not strictly dependent on expression of the DTR transgene. This aligns with previous reports describing similar adverse outcomes in WT and DTR animals. Rubino et al. (2018) documented progressive ataxia-like behaviours accompanied by neuronal loss and reactive gliosis in DT-treated CX3CR1-CreERT2^+^/^−^ROSA26iDTR^+^/^−^ mice. In a follow up study, Peng et al. (2022) reported mortality, microglia activation, motor decline, ataxia-like behaviours, weight loss, and reduced rotarod performance when the same DT regimen (3× 1.0 μg i.p.) was applied to WT mice. This notion also aligns with findings from Bedolla et al. (2022), who reported global cerebrospinal fluid (CSF) loss and ventricular shrinkage in the CX3CR1-iDTR model; although initially attributed to the ROSA26-iDTR allele, comparable changes occurred in DT-treated iDTR^+^ mice lacking the Cre transgene, implicating DT itself as a contributing neurotoxic factor. Together, these studies suggest that such phenotypes are not exclusively attributable to targeted depletion but may instead reflect non-specific neurotoxic effects of DT. Our findings extend these interpretations, as both CD11b-DTR and WT mice exhibited sensitivity to DT and the development of behavioural deficits and mortality despite local DT delivery through i.c.v., further supporting a mechanism of DT driving neuroinflammation and neuronal stress.

To characterise region-specific changes in IBA1 + cell populations CD11b-DTR mice we assessed cells within the hippocampus and midbrain. We found that hippocampal numbers remained unchanged across DT doses, although these cells exhibited an activated morphology. In contrast, low-dose DT produced a clear reduction in IBA1 + cells in the midbrain, indicating that depletion occurs to different extents across brain regions. A likely explanation is that DT distribution varies depending on how CSF moves through the ventricles after i.c.v. administration, which targets the lateral ventricles. CSF flows quickly from the lateral ventricle into the third ventricle and down the cerebral aqueduct, which pass directly through the midbrain, and radiotracer studies have shown that CSF reaches midbrain cisterns within minutes, suggesting that midbrain structures, including the substantia nigra, may be exposed to relatively higher local DT concentrations following i.c.v. administration (Ghersi-Egea et al., 1996; Nagaraja et al., 2005). The hippocampus, however, sits within a more separated part of the lateral ventricle, where CSF flow is likely slower and potentially reduces DT exposure. This idea aligns with findings from Wang et al. (2020), who achieved notable microglial loss in the dentate gyrus of the hippocampus when DT was infused into the third ventricle over a prolonged period, likely reflecting both the central anatomical position of the third ventricle and the cumulative effect of sustained DT delivery. Although i.c.v. administration was selected to reduce peripheral exposure to DT, this study did not directly measure systemic DT or peripheral toxin levels. Therefore, we cannot exclude the possibility that centrally administered DT contributes to peripheral effects via CSF–blood exchange or indirect inflammatory signalling. Together, these points highlight that DT delivery into the ventricles can also produce region-specific effects and further reiterates the need for careful dose regime validation and region-specific assessment in DTR models.

Despite these functional outcomes, our blood and histopathological analyses were largely inconclusive. All peripheral organs appeared normal, and only isolated brain changes were detected in clinically affected animals. The absence of overt systemic pathology suggests that the observed behavioural phenotypes may arise independently of widespread peripheral toxicity and instead reflect direct effects of DT within the brain, consistent with the notion that early biochemical or neuroinflammatory changes can precede detectable histological lesions. In this context, the focal CNS abnormalities observed in the DT-treated animals—including ventriculitis, meningoencephalitis, spongiotic changes, and scattered neuronal necrosis—likely represent early or evolving neuronal dysfunction rather than widespread tissue injury. Although isolated abnormalities were observed in individual DT-treated animals, these findings were not reproducible across groups; for example, neutropenia was detected in only a single DT-treated CD11b-DTR animal, precluding firm conclusions regarding its biological relevance or association with DT exposure. Taken together, these data indicate that overt systemic pathology was not a consistent feature of DT treatment under the conditions tested, although subtle or transient changes cannot be excluded.

To more fully define the mechanisms underlying DT-induced toxicity and region-specific myeloid cell responses, future studies would benefit from incorporating quantitative myeloid cell morphometric analyses, assessment of activation- and homeostatic-state markers beyond IBA1, and complementary approaches such as flow cytometry to distinguish resident microglia from infiltrating myeloid cells. In parallel, serum biochemistry, cytokine profiling, and synaptic or electrophysiological assays may provide more sensitive readouts of early neuronal and inflammatory changes to help clarify how DT contributes to the behavioural phenotypes observed across both genotypes.

Together, our findings highlight that DT exposure, independent of DTR expression, can induce behavioural changes and affect mortality, underscoring the need for rigorous characterisation and dose validation before utilising DTR models. Although DTR systems remain powerful tools for interrogating myeloid cell function *in vivo*, our findings indicate that DT exposure is associated with focal CNS changes and behavioural impairment in both WT and CD11b-DTR mice. Importantly, this demonstrates that behavioural outcomes attributed to myeloid cell depletion may instead reflect DTR-driven toxicity, reinforcing the recommendation that studies aiming to assess behavioural phenotypes must include thorough behavioural testing and appropriate DT-treated controls to verify that observed effects are genuinely cell-specific. Incorporating complementary approaches—such as biochemical markers of neuronal stress, cytokine profiling, and synaptic or circuit-level analyses—will be also essential for distinguishing true cell contributions from DT-driven artefacts. Collectively, these insights reshape how DT-based depletion models should be interpreted and highlight the need for deeper methodological rigor to enhance their translational value and ensure that conclusions about macrophage and microglial function in health and disease are accurately grounded.

## Data Availability

The original contributions presented in the study are included in the article/Supplementary material, further inquiries can be directed to the corresponding author/s.
