# Supplementary material for: Intracerebroventricular diphtheria toxin causes off-target toxicity in CD11b-DTR and wild-type mice, revealing limitations of DTR-based depletion studies
Source: Front Neurosci. 2026 Apr 23;20:1806305. doi: 10.3389/fnins.2026.1806305 (PMC13149294; doi:10.3389/fnins.2026.1806305)
Supplement: Supplementary file 1 [file Supplementary_file_1.docx]

**Supplemental material**


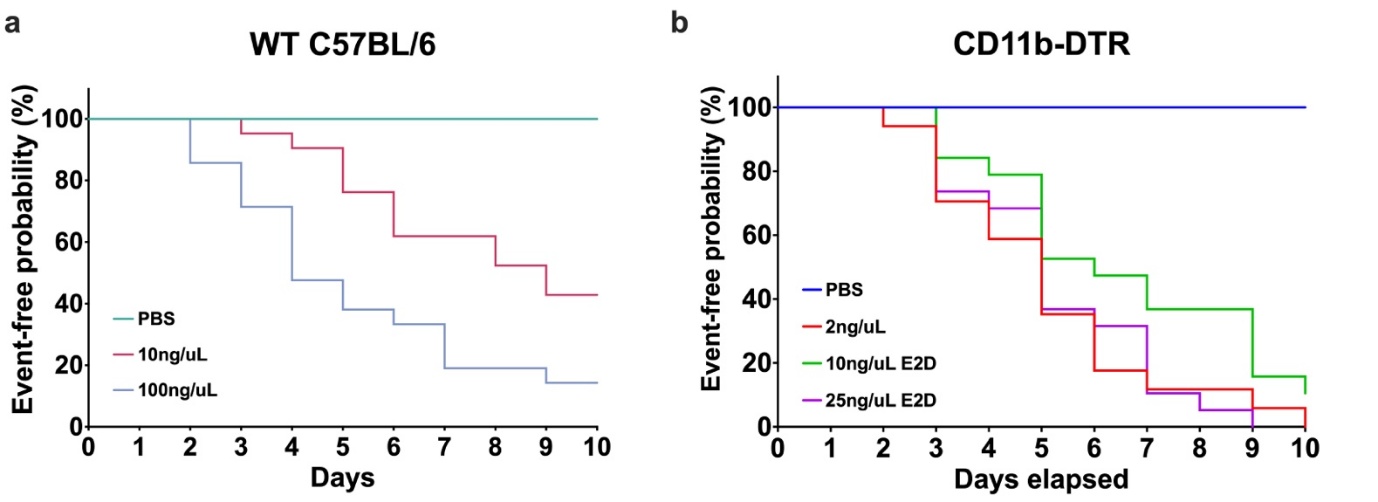


**Supplementary 1 – Development of side-effects**

Kaplan-Meier curves showing time to first observed side effect for (a) WT and (b) CD11b-DTR mice over the 10-day treatment period. Each event represents the first observed occurrence of side effects (ataxia, kyphosis and/or seizure) in an individual animal.

In WT mice, event-free curves differed significantly between treatment groups (log-rank Mantel-Cox test, [χ² = 45.27, df = 2, *p* < 0.0001], *n* = 18-21 per group). At the end of the observation period, 100% of PBS treated mice remained unaffected compared to 33.3% and 0% in the 10ng/µL and 100ng/µL treated groups, respectively.

Similarly, in CD11b-DTR mice, event-free curves were significantly different across groups [χ² = 41.69, df = 2, *p* < 0.0001, *n* = 13-17 per group]. The proportion of animals remaining free from side-effects was 100% in PBS treated mice, compared to 0%, 10.5% and 0% in the 2ng/µL, 10ng/µL and 25ng/µL E2D groups, respectively.

**
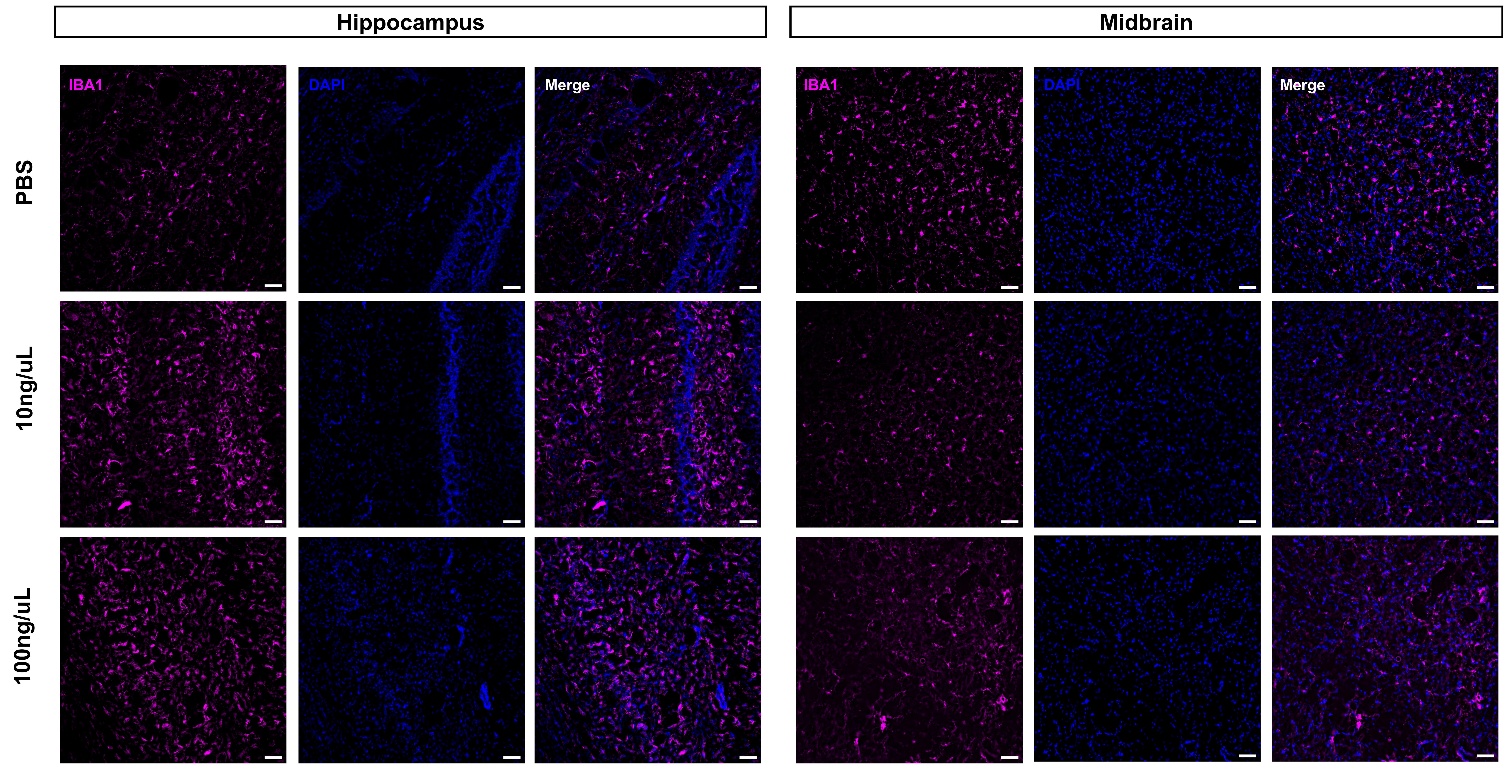
Supplementary 2 – DT-associated changes in cell morphology of IBA1+ cells in C57BL/6 Mice**

Representative images showing IBA1 (magenta) and DAPI (blue) staining in the hippocampus and midbrain following a 10-day i.c.v. administration course of PBS, 10 ng/µL DT, or 100 ng/µL DT in C57BL/6 mice. Images illustrate region- and dose-dependent alterations in IBA1+ myeloid cell morphology, suggestive of a pro-inflammatory state. Scale bar represents 50 μm.

**
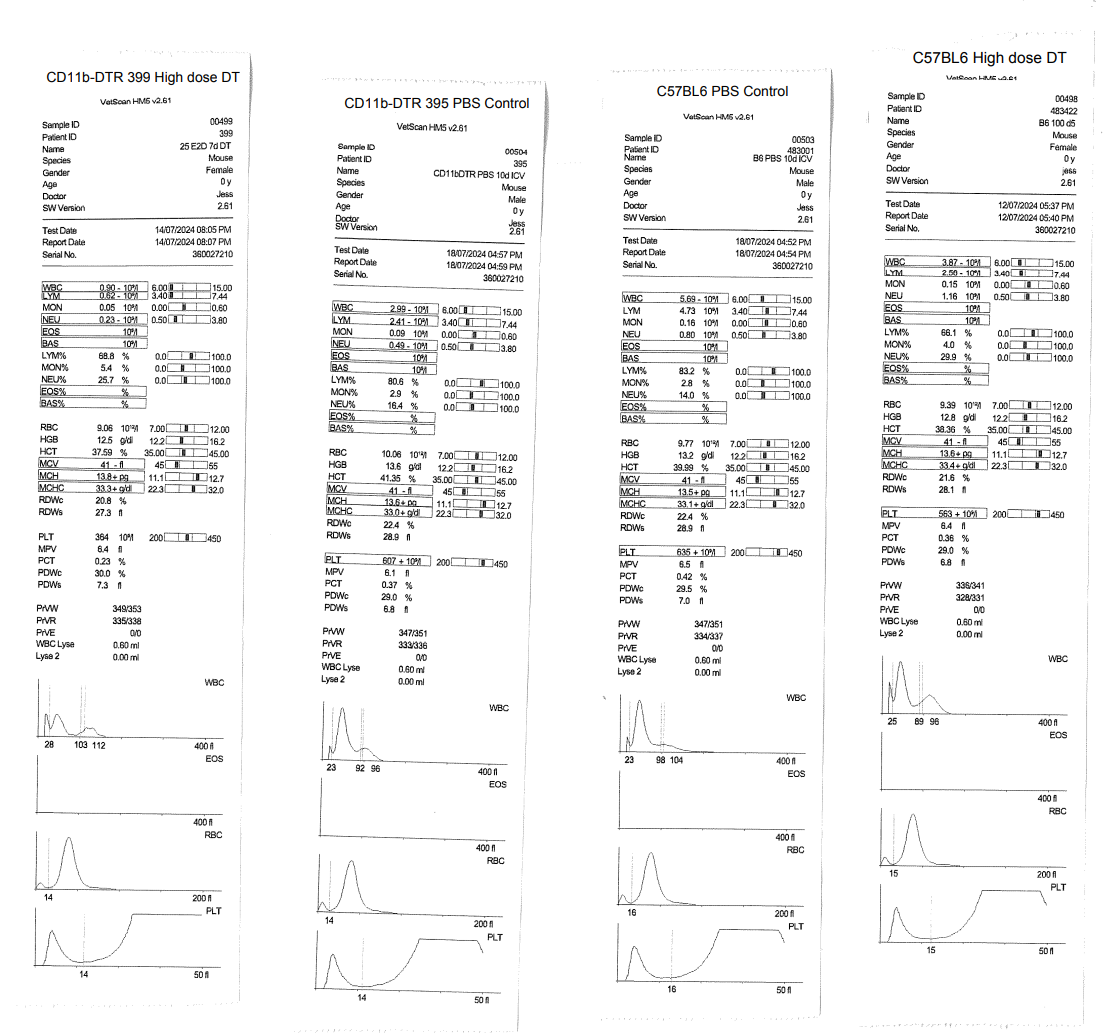
**

**Supplementary 3 – Haematology reports for DT treated CD11b-DTR and C57BL/6 mice**

Full blood count reports from representative CD11b-DTR and C57BL/6 mice treated with PBS or high-dose DT (CD11b-DTR - 25ng/uL/E2D, C57BL/6 – 100ng/uL) are shown to illustrate peripheral haematological profiles. These data are presented to inform potential systemic responses to DT exposure. Haematological analyses were conducted on DT-treated animals exhibiting clinical signs during the DT administration period.

**
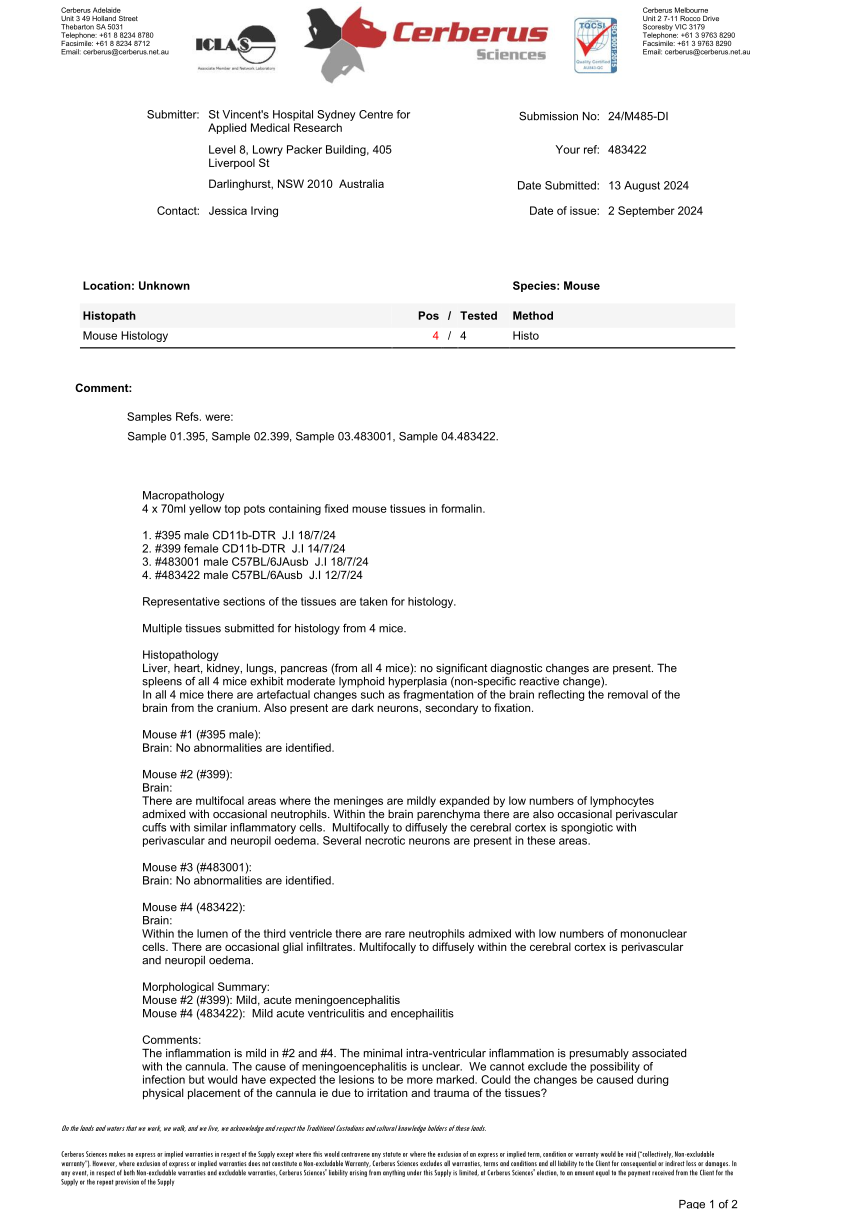
**

**
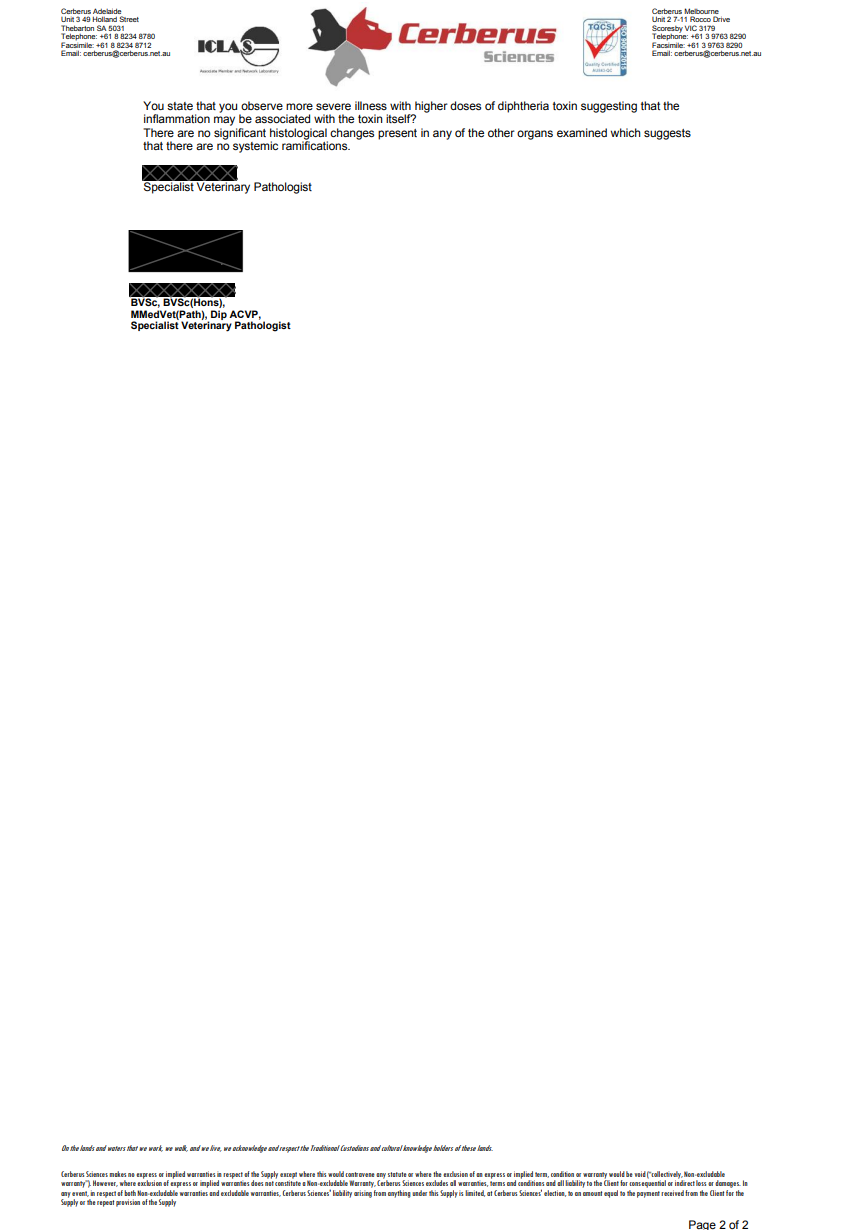
**

**Supplementary 4 – Histopathology findings for DT treated CD11b-DTR and C57BL/6 mice**

Histology report from representative CD11b-DTR and C57BL/6 mice treated with PBS or high-dose DT (Mouse #1: CD11b-DTR PBS, Mouse #2: 25ng/uL E2D, Mouse #3: C57BL/6 PBS, Mouse #4: C57BL/6 100ng/uL). Histological analyses were conducted on DT-treated animals exhibiting clinical signs during the DT administration period.
